# Metabonomic-Transcriptome Integration Analysis on Osteoarthritis and Rheumatoid Arthritis

**DOI:** 10.1155/2020/5925126

**Published:** 2020-01-02

**Authors:** Ningyang Gao, Li Ding, Jian Pang, Yuxin Zheng, Yuelong Cao, Hongsheng Zhan, Yinyu Shi

**Affiliations:** ^1^Shi's Center of Orthopedics and Traumatology, Shuguang Hospital Affiliated to Shanghai University of TCM, No. 528 Zhangheng Road, Shanghai 201203, China; ^2^Institute of Traumatology & Orthopedics, Shanghai Academy of TCM, No. 528 Zhangheng Road, Shanghai 201203, China

## Abstract

**Purpose:**

This study is aimed at exploring the potential metabolite/gene biomarkers, as well as the differences between the molecular mechanisms, of osteoarthritis (OA) and rheumatoid arthritis (RA).

**Methods:**

Transcriptome dataset GSE100786 was downloaded to explore the differentially expressed genes (DEGs) between OA samples and RA samples. Meanwhile, metabolomic dataset MTBLS564 was downloaded and preprocessed to obtain metabolites. Then, the principal component analysis (PCA) and linear models were used to reveal DEG-metabolite relations. Finally, metabolic pathway enrichment analysis was performed to investigate the differences between the molecular mechanisms of OA and RA.

**Results:**

A total of 976 DEGs and 171 metabolites were explored between OA samples and RA samples. The PCA and linear module analysis investigated 186 DEG-metabolite interactions including Glycogenin 1- (GYG1-) asparagine_54, hedgehog acyltransferase- (HHAT-) glucose_70, and TNF receptor-associated factor 3- (TRAF3-) acetoacetate_35. Finally, the KEGG pathway analysis showed that these metabolites were mainly enriched in pathways like gap junction, phagosome, NF-kappa B, and IL-17 pathway.

**Conclusions:**

Genes such as HHAT, GYG1, and TRAF3, as well as metabolites including glucose, asparagine, and acetoacetate, might be implicated in the pathogenesis of OA and RA. Metabolites like ethanol and tyrosine might participate differentially in OA and RA progression via the gap junction pathway and phagosome pathway, respectively. TRAF3-acetoacetate interaction may be involved in regulating inflammation in OA and RA by the NF-kappa B and IL-17 pathway.

## 1. Introduction

Osteoarthritis (OA) and rheumatoid arthritis (RA) are all inflammatory joint diseases [1]. Globally, approximately 250 million people (3.6% of the population) have OA [2]. Meanwhile, RA will affect about 24.5 million people by the year 2015 [3, 4]. Although some factors including cytokines and chemokines help distinguish OA from RA [5], the unclear differences in the molecular mechanisms underlying these two diseases impede the choice of the optimal clinical treatment strategy.

Metabolites have various functions including signaling, defense, and interactions with other organisms [6]. Numerous studies have shown that metabolites are associated with the pathological process of OA and RA [7–9]. A previous study shows that the expression levels of reactive oxygen metabolites are upregulated in patients with knee OA [10]. Zhang et al. indicated that a total of 14 metabolites extracted could be potentially used as biomarkers for OA [11]. Moreover, as a biomarker of in vivo mast cell activation, the activity of prostaglandin D2 metabolites is closely associated with the progression of RA [12]. In an animal model, Jahreis et al. showed that mold metabolites drive RA in mice via the promotion of T cells [13]. However, the interpretation of metabonomic data is difficult due to the obstacle in data extraction and disease correlation analysis [14]. A previous study indicates that the expression of interleukin 1 can regulate the progression of both OA and RA through direct stimulation of synoviocytes and augmentation of matrix degradation [15]. Actually, the detection and interpretation of metabolite-transcript coresponses using combined profiling can yield important information on the complex biological regulation mechanism of the disease [16]. Thus, integrative analysis of transcriptome and metabonomic data may contribute to a further understanding of OA and RA progression.

In the present study, a metabonomic-transcriptome integration analysis was performed. To investigate the genes and metabolites that differentially expressed between OA and RA, the differentially expressed genes (DEGs) and metabolites were revealed from microarray data and metabolite expression profile data, respectively. Then, principal component analysis (PCA) and the linear model were used to explore the DEG-metabolite interactions. Finally, based on these interactions, pathway analysis was performed on DEG-associated metabolites to reveal the pathways that participate in the process of OA and RA. This study was expected to investigate the role of key metabolites and genes as well as their interactions in the pathogenesis of OA and RA, and further understand the complex biological regulatory mechanisms of metabolites in these two diseases.

## 2. Materials and Methods

### 2.1. Data Resource

The transcriptome dataset GSE100786 was downloaded from the Gene Expression Omnibus (GEO) database. The platform was the GPL570 Affymetrix Human Genome U133 Plus 2.0 Array. A total of 8 OA bone marrow (BM) monocyte samples and 8 RA BM monocyte samples were included in GSE100786.

The metabolite profiling MTBLS564 was downloaded from the Ensembl-European Bioinformatics Institute (EMBL-EBI) MetaboLights database (https://www.ebi.ac.uk/metabolights/index). The platform was Bruker (using NMR spectroscopy technology). A total of 10 OA synovial fluid (SF) samples and 14 RA SF samples were included in MTBLS564.

### 2.2. Data Preprocessing

The normalization for transcriptome data was performed using the Robust Multichip Average (RMA) [17] method in the Affy package (version: 1.56.0) [18] of R (version: 3.4.3) software. The normalization process in this study included background adjustment, quantile normalization, and finally summarization and log base 2 scale. If different probes mapped to the same miRNA (miRNA symbol), the mean value of different probes was considered as the final expression value of this miRNA. Meanwhile, the metabolomic data could be directly read from the processed metabolite data file using R software.

### 2.3. The Investigation for DEGs

The *P* value between OA samples and RA samples in transcriptome data was calculated by the Linear Models for Microarray Data (version: 3.34.9, limma) package [19] in R software. Then, *P* < 0.05 was selected as the threshold for the identification of DEGs. Then, based on Euclidean distance, the bidirectional hierarchical clustering for DEMs was performed by pheatmap software (version: 1.0.8) [20]. The results were visualized using a heat map.

### 2.4. Principal Component Analysis

In the current study, the average value of each gene in the transcriptome data was calculated and ranked from high to low (deleting the last 10% of the genes). Meanwhile, the proportion of the deletion value in the expression value of each metabolite was counted, and the metabolites with more than 80% deletion value were deleted. Then, the principal component analysis (PCA) was performed on the data in two groups.

### 2.5. Integrating Transcriptome Data and Metabonomic Data Based on Linear Models

Based on the results of PCA analysis on gene expression data and metabonomic data, the *P* values of DEG-metabolite relevance between OA samples and RA samples were obtained using the IntLim (version: 0.1.0, https://github.com/mathelab/IntLIM) linear model algorithm [21]. The computation formula is as follows:

(1) $$\begin{array}{c} m=\beta 1+\beta 2g+\beta 3p+\beta 4\left(g:p\right)+\varepsilon , \end{array}$$

where “*m*” and “*g*” in the formula represent metabolite abundance and gene expression level, respectively; “*p* ” in the formula represents phenotype (OA samples vs. RA samples); “(*g* : *p*)” in the formula represents the association between gene expression and phenotype; and “*ε*” in the formula represents normal distribution. Then, the difference of correlation coefficients between the two groups (|*r*_OA_ − *r*_RA_|) > 1 and *P* value < 0.001 were selected as the cut-off values for DEG-metabolite interaction investigation.

### 2.6. Metabolic Pathway Enrichment Analysis

The clusterProfiler software (version: 3.2.11) [22] is an online tool that provides enrichment analyses including KEGG [23]. Based on the *P* value of DEG-metabolite interactions, the KEGG pathway enrichment analysis was used to investigate pathways enriched by the DEGs associated with metabolites. *P* value (the significance threshold of the hypergeometric test) < 0.05 was chosen as the cut-off criterion for the present enrichment analysis.

## 3. Results

### 3.1. DEG and Metabolite Investigation

**Figure 1:**
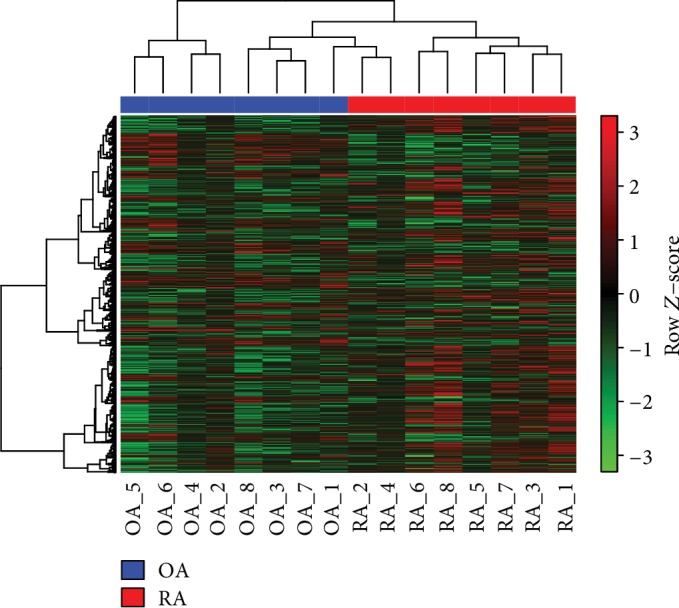
The heat map for differentially expressed genes between osteoarthritis samples and rheumatoid arthritis samples. The blue and red bars at the top represent the samples in osteoarthritis samples and rheumatoid arthritis samples, respectively. The red color represents low expression, while the red color represents high expression.

After preprocessing, a total of 171 metabolites from metabonomic data were enrolled for further investigation. Meanwhile, a total of 20,192 genes were obtained from 54,675 probes in the current transcriptome data. Among these 20,192 genes, a total of 416 upregulated genes (such as hedgehog acyltransferase (HHAT)) and 669 downregulated genes (such as Glycogenin 1 (GYG1), Unc-51 Like Kinase 3 (ULK3), and breakpoint cluster region protein (BCR)) were revealed between OA samples and RA samples in transcriptome data. The heat map for all these DEGs is shown in Figure 1.

### 3.2. DEG-Metabolite Interaction Analysis

**Figure 2:**
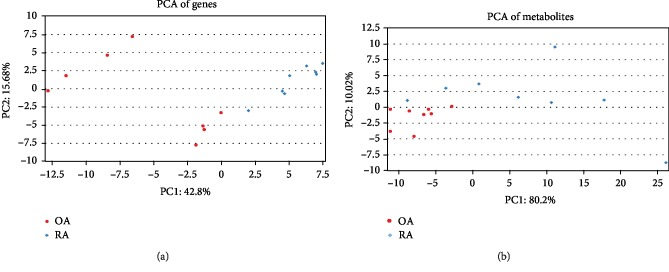
The principal component analysis of metabolites and differentially expressed genes. (a) The principal component analysis for differentially expressed genes in transcriptome data. (b) The principal component analysis for metabolites in metabonomic data. The red circles represent osteoarthritis (OA) samples; the blue diamonds represent the rheumatoid arthritis (RA) samples.

**Figure 3:**
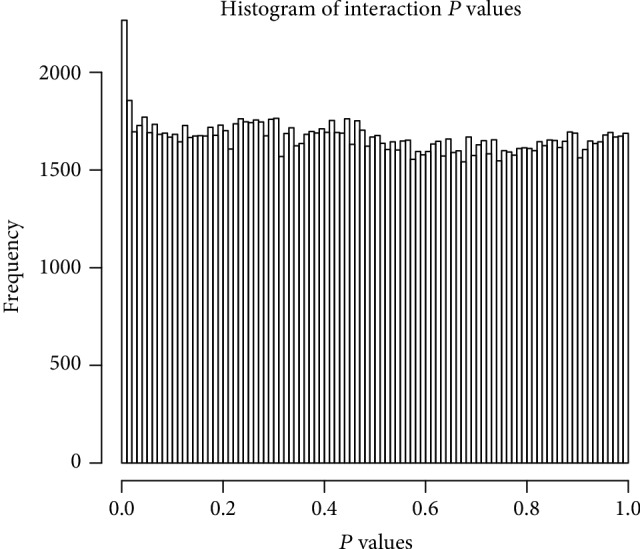
The histogram of interaction *P* values for all differentially expressed gene-metabolite relations in the current study. The *x*-axis represents the *P* value of certain differentially expressed gene-metabolite relations, while the *y*-axis represents the frequency of this *P* value.

**Figure 4:**
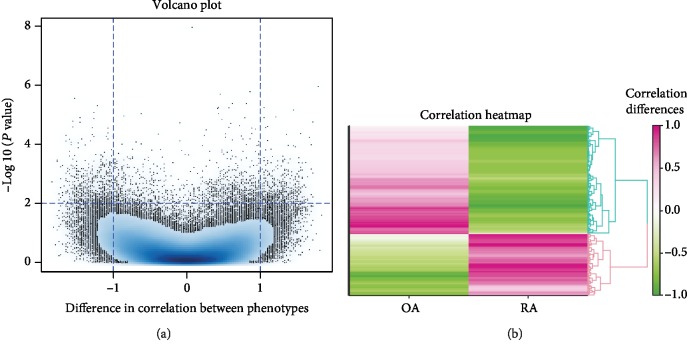
The volcano plot and heat map for correlations between differentially expressed genes and metabolites. (a) The volcano plot: *x*-axis represents the difference in correlation between differentially expressed gene and metabolites; *y*-axis represents the *P* value of correlation. (b) The heat map: OA—the correlation between differentially expressed genes and metabolites in the osteoarthritis group; RA—the correlation between differentially expressed genes and metabolites in the rheumatoid arthritis group; the different colors represent the correlation difference.

**Figure 5:**
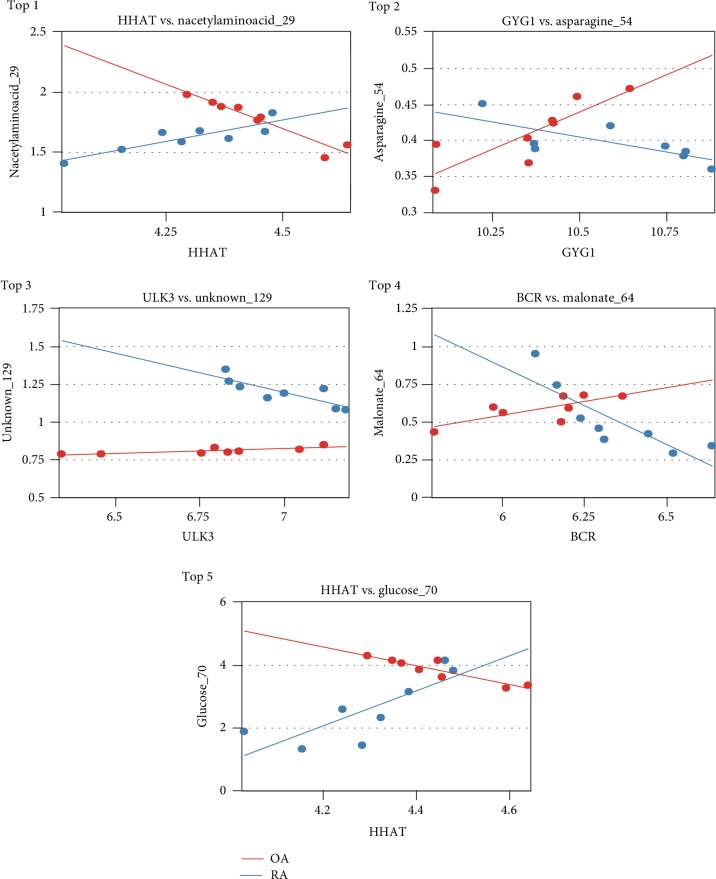
The correlation for the top 5 differentially expressed gene-metabolite relations. The *x*-axis represents the expression of differentially expressed genes; the *y*-axis represents the expression of metabolites. OA: osteoarthritis; RA: rheumatoid arthritis.

After filtering the transcriptome data and metabonomic data, the PCA analysis was performed on 171 metabolites and 976 DEGs. The results of PCA analysis for these metabolites and DEGs are shown in Figure 2. Furthermore, the histogram of *P* values of the DEG-metabolite relevance between OA samples and RA samples is shown in Figure 3. The result of the correlation analysis between all DEGs and metabolites is shown in Figure 4(a). Meanwhile, the correlation analysis between DEGs and metabolites in the OA group or the RA group is shown in Figure 4(b). These results showed that a total of 186 interactions including 98 metabolites and 94 DEGs were revealed among all DEG-metabolite relations with *P* < 0.001, such as TRAF3-acetoacetate_35 (Supplemental [Supplementary-material supplementary-material-1]). According to the value of |*r*_OA_ − *r*_RA_|, the results of the top 5 DEG-metabolite interactions including HHAT-nacetylaminoacid_29, GYG1-asparagine_54, ULK3-unknown_129, BCR-malonate_64, and HHAT-glucose_70 are listed in Table 1. Moreover, the correlation analyses of these 5 interactions are shown in Figure 5.

### 3.3. Enrichment Analysis for Metabolites

**Figure 6:**
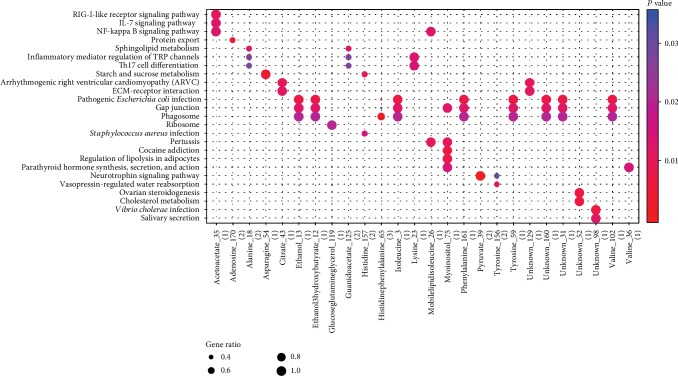
The result of enrichment analysis for metabolites in differentially expressed gene-metabolite interactions. The *x*-axis represents the metabolites; *y*-axis represents the item of pathway. The change from blue to red indicates the correlation from low to high; GeneRatio is the proportion of genes; the larger the proportion of genes, the greater the proportion of enriched items.

The KEGG pathway enrichment analyses were performed on 26 metabolites in DEG-metabolite interactions. The results showed that these metabolites were mainly enriched in pathways like the pathogenic *Escherichia coli* infection pathway (hsa05130; *P* = 1.77*e*‐02; metabolites: ethanol_13, isoleucine_3, tyrosine_59, etc.), gap junction (hsa04540; *P* = 1.77*e*‐02; metabolites: ethanol_13, isoleucine_3, tyrosine_59, etc.), phagosome (hsa04145, *P* = 2.04*e*‐02; metabolites: ethanol_13, isoleucine_3, tyrosine_59, etc.), NF-kappa B signaling pathway (hsa04064; *P* = 1.27*e*‐02; metabolite: acetoacetate_35), IL-17 signaling pathway (hsa04657; *P* = 1.25*e*‐02; metabolite: acetoacetate_35), and inflammatory mediator regulation of TRP channels (hsa04750; *P* = 2.63*e*‐02; metabolites: alanine_18, guanidoacetate_125, and lysine_23) (Figure 6).

## 4. Discussion

Although metabolites are proven to be associated with the pathological process of OA and RA, the difficulty in interpreting metabonomic data impedes the understanding of the differences of the molecular mechanisms between these two diseases. The current metabonomic-transcriptome integration analysis revealed a total of 976 DEGs and 171 metabolites between OA samples and RA samples. The PCA and linear module analysis explored 186 DEG-metabolite interactions including GYG1-asparagine_54, HHAT-glucose_70, and TRAF3-acetoacetate_35. Finally, the KEGG pathway analysis showed that these metabolites were mainly enriched in pathways like pathogenic *Escherichia coli* infection, gap junction, phagosome, NF-kappa B signaling pathway, and IL-17 signaling pathway.

HHAT is an enzyme in the endoplasmic reticulum that palmitoylates hedgehog proteins [24]. HHATs participate in the expression of the sonic hedgehog signaling pathway [25]. The sonic hedgehog signaling pathway can regulate the neuronal-like differentiation of bone mesenchymal stem cells [26]. It also promotes carcinoma cells associated with bone destruction [27]. A previous study shows that sonic hedgehog signaling pathway-associated factors are upregulated in synovial tissues of RA [28]. Wang et al. indicated that genes such as HHAT in the sonic hedgehog signaling pathway are novel therapeutic targets for RA [29]. Interestingly, sonic hedgehog alleviates the inhibitory effects of high glucose on the osteoblastic differentiation of bone marrow stromal cells [30]. Glucose deficiency is closely related with the development of OA [31]. A clinical and epidemiological survey shows that the plasma glucose concentration is associated with the severity of OA [32]. Data from the OA investigation indicates that glucose homeostasis can influence the risk of knee OA. Moreover, GYG1 is an enzyme that is involved in the biosynthesis of glycogen [33]. It plays a role in glycogen metabolism regulation and in the maximal glycogen level attainment in skeletal muscle [34]. A previous study shows that the inhibition of GYG synthase kinase attenuates the glucocorticoid-induced bone loss [35]. A study of hepatic glycogen catabolism and glycogen levels in rats with chronic arthritis shows that GYG expression level is closely related with arthritis progression [36]. Meanwhile, a previous study reveals that asparagine-linked glycosylation of bone morphogenetic protein is required for secretion and osteoblast differentiation [37]. In a clinical study, Tong et al. indicated that asparagine levels were all below the lower limit of quantification in the bone marrow of leukemia patients [38]. In this study, the PCA and linear module analysis showed that GYG1-asparagine and HHAT-glucose were two of the most outstanding DEG-metabolites. Thus, we speculate that GYG1-asparagine and HHAT-glucose interaction might be implicated in the pathogenesis of OA and RA.

A previous study shows that gap junctions have an important function in the control or coordination of bone cell activity [39]. The intercellular gap junctions play a vital role in skeletal physiology and bone cell mechanosensing [40]. The metabolite ethanol decreases gap junction permeability in primary cultures from defined brain regions [41]. Actually, ethanol had been successfully used in the treatment of distal tarsal joint OA [42]. Meanwhile, Jonsson et al. indicated that ethanol could prevent the development of destructive RA [43]. Although the increased intercellular communication through gap junctions may contribute to the progression of OA [44], so far, no study has shown that the gap junction pathway is involved in RA progression. Furthermore, a previous study indicated that phagosome-lysosome fusion participates in the biological function of bone marrow macrophages [45]. Based on a fluorescence and electron microscope study, Zuckerfranklin showed that phagosomes participated in the regulation of synovial fluid leukocytes during RA development [46]. A previous study showed that some metabolites including tyrosine participated in the phagosome pathway [47]. An expression profile analysis of tyrosine genes in human OA proves the relationship between tyrosine expression and OA progression [48]. Meanwhile, Murakami et al. indicated that the tyrosine kinase promotes the development of RA through the activation of macrophages [49]. In this study, KEGG pathway analysis showed that the DEG-associated metabolites between OA samples and RA samples such as ethanol and tyrosine were mainly enriched in pathways like the gap junction and phagosome, respectively. Thus, we speculated that the gap junction pathway enriched by the ethanol and phagosome pathway enriched by tyrosine might participate differentially in OA and RA progression.

Studies had reported the roles of NF-kappa B and IL-17 on regulating inflammation in RA [50–52]. Shui et al. suggested that inflammation could be alleviated by blocking IL-17 in RA rats [53]. Zhang et al. indicated that overexpression of microRNA-125b could facilitate inflammation in RA by activating the NF-*κ*B signaling pathway [54]. In this study, metabolite acetoacetate was significantly enriched in the NF-kappa B and IL-17 signaling pathway, and TRAF3-acetoacetate_35 interaction was identified. TRAF3 is an intracellular protein that belongs to the TNF receptor-associated factor protein family which is implicated in NF-kappa B activation [55, 56]. Reportedly, the Tengmei Decoction could improve inflammatory injury of synovium in collagen-induced arthritis rats probably by regulating the TRAF3/NF-*κ*B signaling pathway [57]. Liu et al. showed that miR-671-3p played a crucial role in the pathogenesis of OA by targeting TRAF3 and regulating chondrocyte apoptosis and inflammation [58]. Thereby, we speculated that the NF-kappa B and IL-17 pathway enriched by acetoacetate was implicated in regulating inflammation in OA and RA probably by targeting TRAF3. However, there were some limitations in the current study including small sample size and lack of verification analysis. Thus, a further verification study based on a large sample size is needed to confirm all speculations in this study.

## 5. Conclusions

In conclusion, genes such as HHAT, GYG1, and TRAF3, as well as metabolites including glucose, asparagine, and acetoacetate might be implicated in the pathogenesis of OA and RA. Metabolites like ethanol and tyrosine may participate differentially in OA and RA progression via the gap junction pathway and the phagosome pathway, respectively. TRAF3-acetoacetate interaction may be involved in regulating inflammation in OA and RA by the NF-kappa B and IL-17 pathway.

## Figures and Tables

**Table 1:** The top 5 differentially expressed gene-metabolite interactions.

Metabolites	DEGs	OA_cor	RA_cor	diff.corr	*P*
Nacetylaminoacid_29	HHAT	-0.952	0.833	1.786	1.12*e*‐06
Asparagine_54	GYG1	0.905	-0.857	-1.762	3.77*e*‐04
Unknown_129	ULK3	0.857	-0.905	-1.762	3.40*e*‐04
Malonate_64	BCR	0.762	-0.952	-1.714	5.83*e*‐04
Glucose_70	HHAT	-0.905	0.810	1.714	9.34*e*‐04

Notes: DEGs: differentially expressed genes; OA_cor: the correlation between DEGs and metabolites in the osteoarthritis group; RA_cor: the correlation between DEGs and metabolites in rheumatoid arthritis correlation; diff.corr: the DEG-metabolite correlation between the OA group and the RA group; *P* < 0.001 was selected as the cut-off value.

## Data Availability

The datasets generated and/or analyzed during the current study are available from the corresponding author on reasonable request.

## Supplementary Materials

Supplementary Materials The obtained DEG-metabolite interactions with *P* < 0.001.
